# Supplementary material for: Can HER2 1+ Breast Cancer Be Considered as HER2-Low Tumor? A Comparison of Clinicopathological Features, Quantitative HER2 mRNA Levels, and Prognosis among HER2-Negative Breast Cancer
Source: Cancers (Basel). 2022 Aug 31;14(17):4250. doi: 10.3390/cancers14174250 (PMC9455006; doi:10.3390/cancers14174250)
Supplement: Supplementary file 1 [file cancers-14-04250-s001.zip › cancers-1884313-supplementary.pdf]

Supplementary Materials

Can HER2 1+ Breast Cancer Be Considered as HER2-Low Tumor? A Comparison of Clinicopathological Features, Quantitative HER2 mRNA Levels, and Prognosis among HER2-Negative Breast Cancer

Lan Shu †, Yiwei Tong †, Zhuoxuan Li, Xiaosong Chen * and Kunwei Shen *

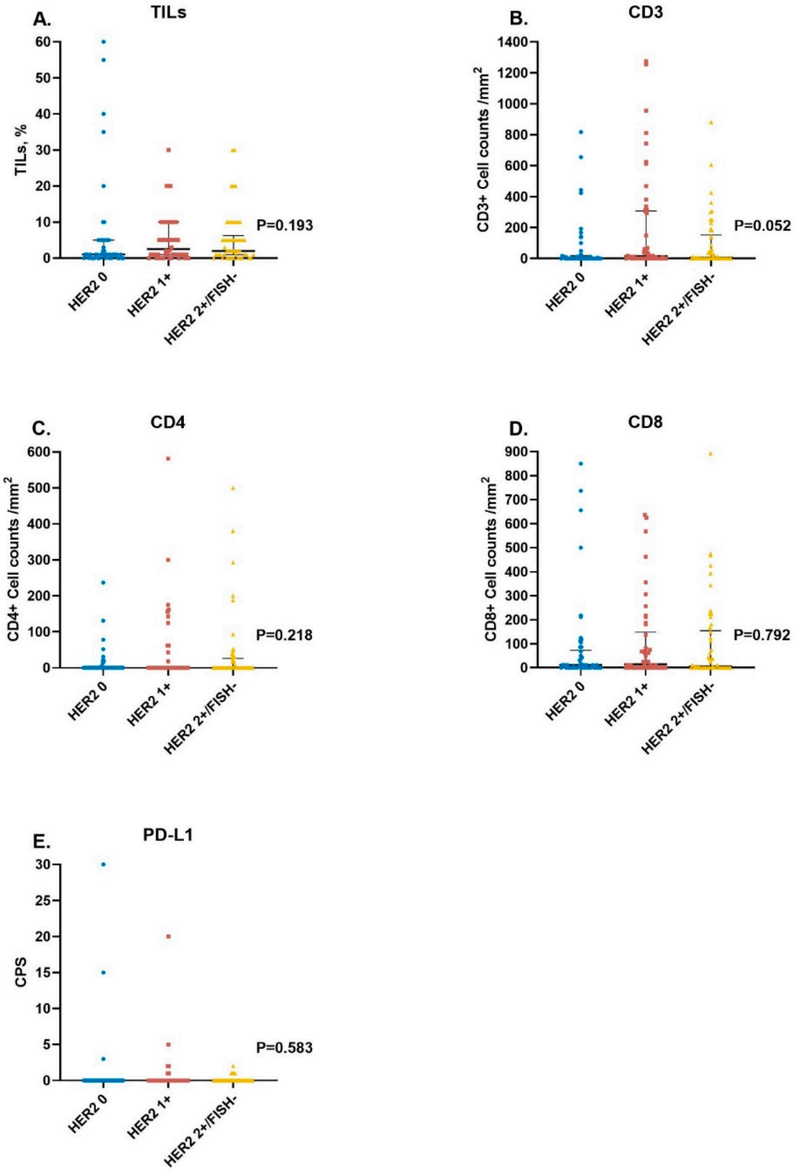

Figure S1. Distribution of tumor immune microenvironment markers by HER2 IHC/FISH status. Counts of stromal TILs, CD3+, CD4+, CD8+ cells, and CPS of PD-L1 were compared using Kruskal-Wallis test among HER2 0, 1+, and 2+/FISH− groups. Solid line represents 25% percentile, median, and 75% percentile. *p* values were reported beside each box. Abbreviation: HER2, human epidermal growth factor receptor 2; IHC, immunohistochemistry; FISH, fluorescence in-situ hybridization; TILs, tumor infiltrating lymphocytes; CPS, combined positive score.

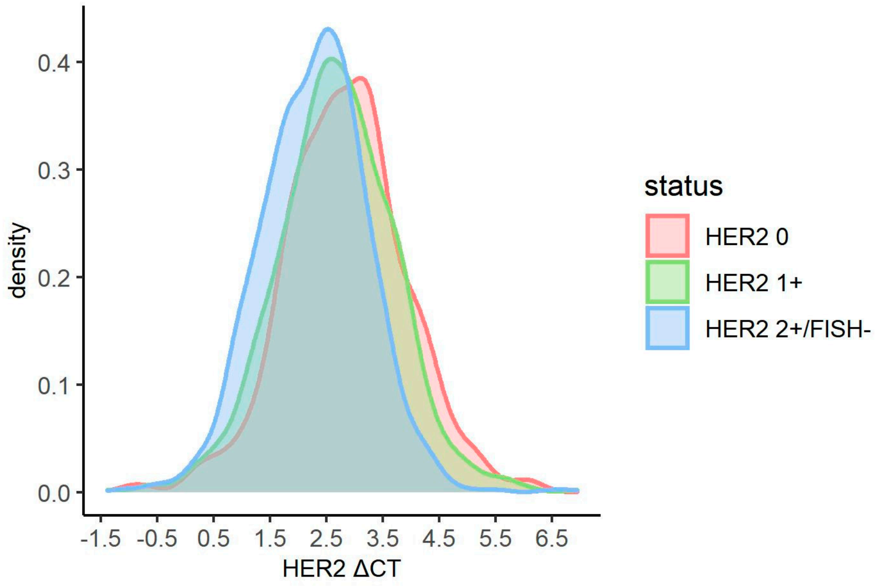

Figure S2. Smoothed density plot of HER2 mRNA expression by different HER2 IHC/FISH groups. Significant overlaps were shown between HER2 0 and HER2 1+ groups, relatively distinct from HER2 2+/FISH− group. Abbreviation: HER2, human epidermal growth factor receptor 2; mRNA, message ribonucleic acid; IHC, immunohistochemistry; FISH, fluorescence in-situ hybridization; CT, cycle threshold.

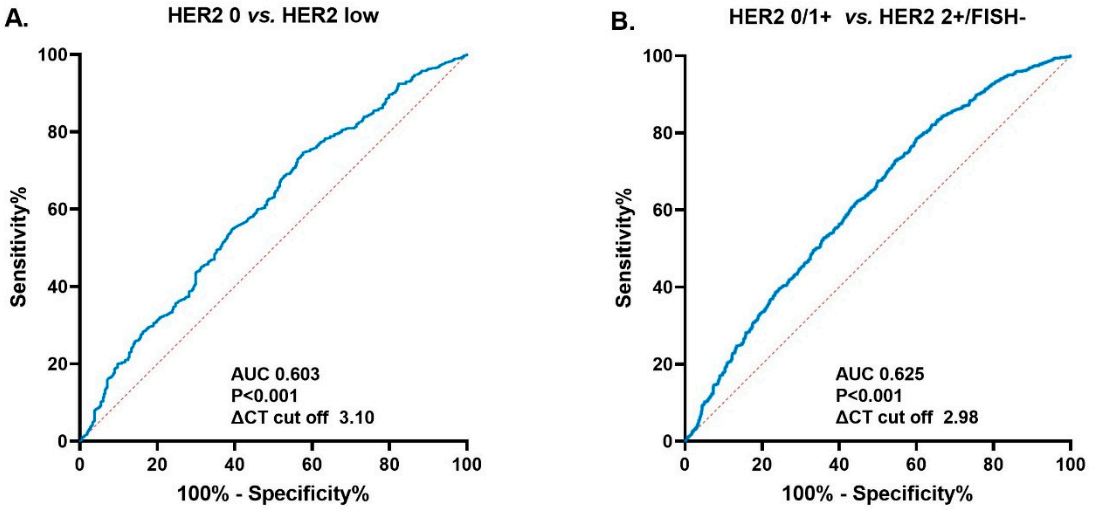

Figure S3. ROC curve and cut-off values for ΔCT in different HER2 IHC/FISH statuses. The ΔCT cut-off was obtained by ROC at the largest Youden index. (**A**) The ΔCT cut-off is 3.10 between HER2 0 and HER2-low cases (AUC 0.603, p<0.001); (**B**) The ΔCT cut-off is 2.98 between HER2 0/1+ and HER2 2+/FISH− cases (AUC 0.603, p<0.001). Abbreviation: HER2, human epidermal growth factor receptor 2; IHC, immunohistochemistry; FISH, fluorescence in-situ hybridization; mRNA, message ribonucleic acid; CT, cycle threshold; ROC, receiver operating characteristic; AUC, area under the curve.

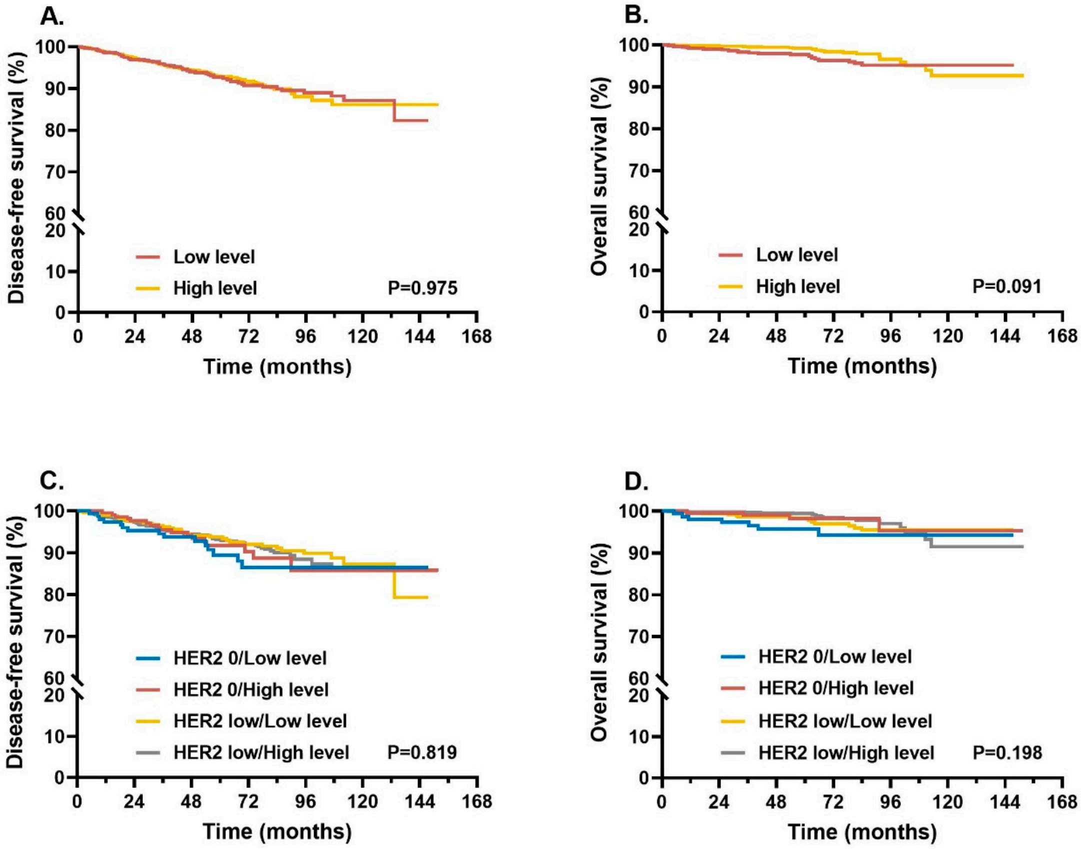

Figure S4. Survival by HER2 mRNA levels [a] in HER2 0 and HER2-low patients. Kaplan–Meier curves of DFS (**A**) and OS (**B**) were compared between low level and high level patients categorized by HER2 mRNA expression in the whole population. Further comparison of DFS (**C**) and OS (**D**) was done according to HER2 IHC status and mRNA expression levels. *p* values for log-rank tests were reported at the bottom of each box. [a] The ΔCT cut-off between HER2 0 and HER2-low was 3.10 according to the ROC curve (low level, ΔCT≥ 3.10; high level, ΔCT< 3.10). Abbreviation: HER2, human epidermal growth factor receptor 2; IHC, immunohistochemistry; FISH, fluorescence in-situ hybridization; DFS, disease-free survival; OS, overall survival; CT, cycle threshold; ROC, receiver operating characteristic.

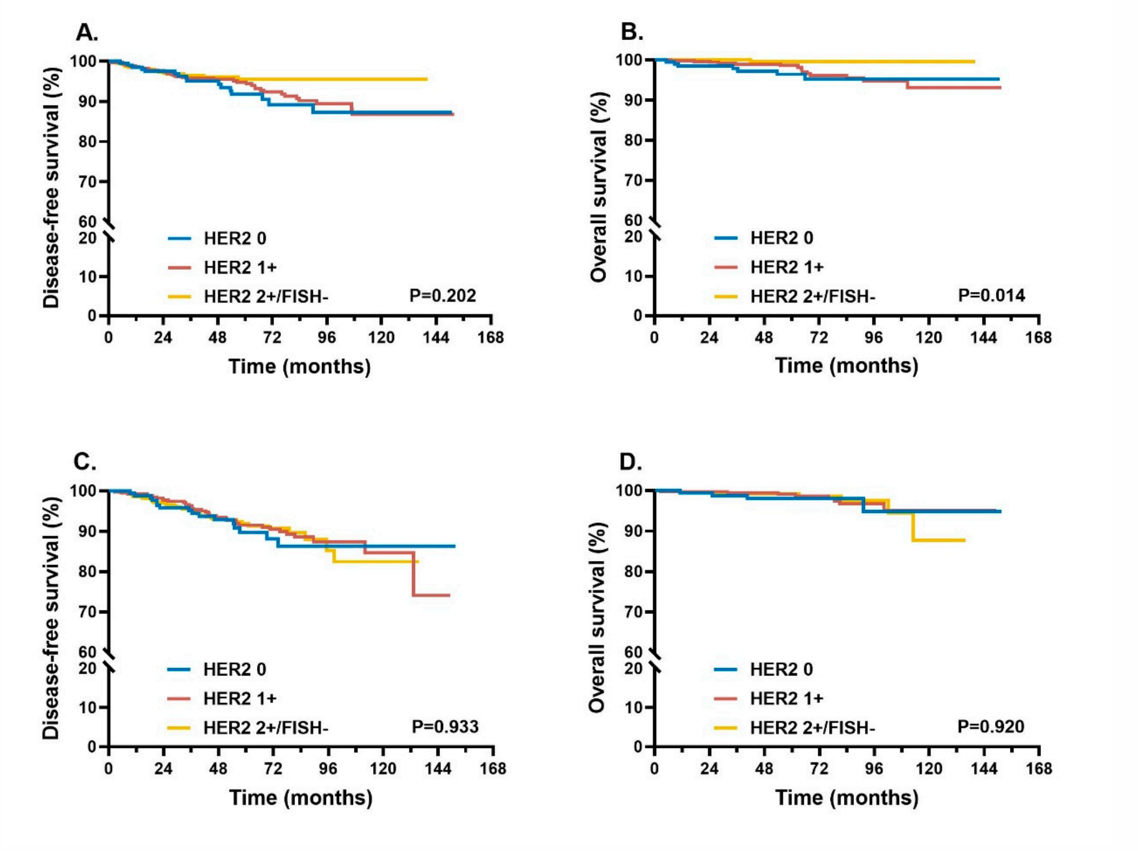

Figure S5. Survival comparison among HER2 0, HER2 1+ and HER2 2+/FISH− patients in chemotherapy-treated and chemotherapy-free cohorts. Kaplan-Meier curves of DFS (**A**) and OS (**B**) were compared among HER2 0, HER2 1+, HER2 2+/FISH− cases in the population who did not received chemotherapy. Kaplan–Meier curves of DFS (**C**) and OS (**D**) were compared among HER2 0, HER2 1+, HER2 2+/FISH− cases in the population who received chemotherapy. *p* values for log-rank tests were reported at the bottom of each box. Abbreviation: HER2, human epidermal growth factor receptor 2; FISH, fluorescence in-situ hybridization; DFS, disease-free survival; OS, overall survival.

Table S1. Tumor immune microenvironment markers by HER2 IHC/FISH status.

Characteristics	Total *n* =150 (%)	HER2 0 *n* = 50(%)	HER2 1+ *n* =50(%)	HER2 2+/FISH− *n* =50 (%)	*p*
TILs (%)					0.292
Mean ± SD	5.5 ± 9.4	6.0 ± 13.2	5.1 ± 6.4	5.4 ± 7.2	
<10	118 (78.7)	43 (86.0)	37 (74.0)	38 (76.0)	
≥10	32 (21.3)	7 (14.0)	13 (26.0)	12 (24.0)	
CD3+ (cell /mm^2)					0.121
Mean ± SD	112.3 ± 238.5	65.1 ± 167.9	185.6 ± 327.8	97.1 ± 175.5	
≤3	73 (48.7)	30 (60.0)	20 (40.0)	23 (46.0)	
>3	77 (51.3)	20 (40.0)	30 (60.0)	27 (54.0)	
CD4+ (cell /mm^2)					0.220
Mean ± SD	29.5 ± 85.3	12.1 ± 39.7	36.5 ± 99.8	39.7 ± 100.7	
0	112 (74.7)	40 (80.0)	39 (78.0)	33 (66.0)	
>0	38 (25.3)	10 (20.0)	11 (22.0)	17 (34.0)	
CD8+ (cell /mm^2)					0.601
Mean ± SD	99.2 ± 189.0	83.6 ± 190.1	114.4 ± 202.1	99.8 ± 176.5	
≤12	70 (46.7)	23 (46.0)	21 (42.0)	26 (52.0)	
>12	80 (53.3)	27 (54.0)	29 (58.0)	24 (48.0)	
PD-L1					0.513

Mean ± SD	0.6 ± 3.2	1.0 ± 4.7	0.6 ± 2.9	0.2 ± 0.4
CPS <1	135 (90.0)	47 (94.0)	44 (88.0)	44 (88.0)
CPS ≥1	15 (10.0)	3 (6.0)	6 (12.0)	6 (12.0)

Abbreviation: HER2, human epidermal growth factor receptor 2; IHC, immunohistochemistry; FISH, fluorescence in-situ hybridization; TILs, tumor infiltrating lymphocytes; SD, standard deviation; CPS, combined positive score.

Table S2. Status of adjuvant therapy by HER2 IHC/FISH status.

Characteristics	Total *n* = 2296 (%)	HER2 0 *n* = 368 (%)	HER2 1+ *n* = 911 (%)	HER2 2+/FISH− *n* = 1017 (%)	*p* value
Chemotherapy					<0.001
Yes	1141 (49.7)	171 (46.5)	408 (44.8)	562 (55.3)	
No	1155 (50.3)	197 (53.5)	503 (55.2)	455 (44.7)	
Radiation therapy					0.954
Yes	1123 (48.9)	181 (49.2)	442 (48.5)	500 (49.2)	
No	1173 (51.1)	187 (50.8)	469 (51.5)	517 (50.8)	
Endocrine therapy					0.112
Yes	2230 (97.1)	352 (95.7)	891 (97.8)	987 (97.1)	
No	66 (2.9)	16 (4.3)	20 (2.2)	30 (2.9)	

Abbreviation: HER2, human epidermal growth factor receptor 2; IHC, immunohistochemistry; FISH, fluorescence in-situ hybridization.

Table S3. Univariate analysis of prognostic factors in HR+/HER2− tumors

Predictors	DFS	OS
	p	*p*
Age (<60 y/o vs. ≥60 y/o)	0.192	0.226
Gender (Female vs. Male)	0.235	0.551
Histology (IDC vs. Non-IDC)	0.490	0.802
TNM stage (I vs. II vs. III)	<0.001	0.011
Tumor size (<2cm vs. ≥2cm)	<0.001	0.008
Breast surgery (BCS vs. Mastectomy)	0.349	0.037
ALN status (negative vs. positive)	0.583	0.471
Histological grade (I-II vs. III)	0.005	0.023
ER (negative vs. positive)	0.510	0.510
PR (negative vs. positive)	0.031	0.109
HER2 (0 vs. 1+ vs. 2+/FISH−)	0.613	0.119
Ki-67 (<14% vs. ≥14%)	0.003	0.169
Chemotherapy (Yes vs. No)	0.030	0.836

Abbreviation: HR, hormone receptor; HER2, human epidermal growth factor receptor 2; y/o, years old; IDC, invasive ductal carcinoma; BCS, breast-conserving surgery; ALN, axillary lymph node; ER, estrogen receptor; PR, progesterone receptor; FISH, fluorescence in-situ hybridization; DFS, disease-free survival; OS, overall survival.

Table S4. Multivariate analysis of prognostic factors in HR+/HER2− tumors.

Characteristics	DFS			OS		
	Hazard Ratio	95% CI	*p*	Hazard Ratio	95% CI	*p*
Tumor Size			<0.001			0.024
<2cm	0.55	0.40–0.76		0.48	0.25–0.91	
≥2cm	1.00			1.00		
Breast surgery			0.156			0.938
BCS	1.40	0.88–2.24		0.96	0.37–2.49	
Mastectomy	1.00			1.00		
Histological grade			0.154			0.041
I-II	0.76	0.52–1.10		0.49	0.24–0.97	

III	1.00			1.00		
PR			0.172			0.240
Negative	1.34	0.88–2.03		1.57	0.74–3.32	
Positive	1.00			1.00		
HER2 status			0.496			0.107
0	1.29	0.84–2.00		2.53	1.03–6.20	
1+	1.14	0.81–1.63		2.00	0.91–4.35	
2+/FISH−	1.00			1.00		
Ki-67			0.148			0.490
<14%	0.77	0.54–1.10		0.78	0.39–1.57	
≥14%	1.00			1.00		
Chemotherapy			0.394			0.306
Yes	1.17	0.82–1.67		0.70	0.36–1.38	
No	1.00			1.00		

Abbreviation: HR, hormone receptor; HER2, human epidermal growth factor receptor 2; BCS, breast-conserving surgery; PR, progesterone receptor; FISH, fluorescence in-situ hybridization; DFS, disease-free survival; OS, overall survival; CI, confidence interval.
